# Differentiation between non-small cell lung cancer and radiation pneumonitis after carbon-ion radiotherapy by ^18^F-FDG PET/CT texture analysis

**DOI:** 10.1038/s41598-021-90674-w

**Published:** 2021-06-01

**Authors:** Makito Suga, Ryuichi Nishii, Kenta Miwa, Yuto Kamitaka, Kana Yamazaki, Kentaro Tamura, Naoyoshi Yamamoto, Ryosuke Kohno, Masato Kobayashi, Katsuyuki Tanimoto, Hiroshi Tsuji, Tatsuya Higashi

**Affiliations:** 1 Department of Molecular Imaging and Theranostics, National Institute of Radiological Sciences, QST, 4-9-1 Anagawa, Inage-ku, Chiba, 263-8555 Japan; 2 Division of Radiation Therapy Technology, Department of Medical Technology, Kanagawa Cancer Center, Kanagawa, Japan; 3 QST Hospital, National Institutes for Quantum and Radiological Science and Technology (QST), Chiba, Japan; 4 Department of Radiological Sciences, School of Health Science, International University of Health and Welfare, Tochigi, Japan; 5 School of Health Sciences, Institute of Medical, Pharmaceutical and Health Sciences, Kanazawa University, Ishikawa, Japan

**Keywords:** Medical research, Oncology

## Abstract

The differentiation of non-small cell lung cancer (NSCLC) and radiation pneumonitis (RP) is critically essential for selecting optimal clinical therapeutic strategies to manage post carbon-ion radiotherapy (CIRT) in patients with NSCLC. The aim of this study was to assess the ability of ^18^F-FDG PET/CT metabolic parameters and its textural image features to differentiate NSCLC from RP after CIRT to develop a differential diagnosis of malignancy and benign lesion. We retrospectively analyzed ^18^F-FDG PET/CT image data from 32 patients with histopathologically proven NSCLC who were scheduled to undergo CIRT and 31 patients diagnosed with RP after CIRT. The SUV parameters, metabolic tumor volume (MTV), total lesion glycolysis (TLG) as well as fifty-six texture parameters derived from seven matrices were determined using PETSTAT image-analysis software. Data were statistically compared between NSCLC and RP using Wilcoxon rank-sum tests. Diagnostic accuracy was assessed using receiver operating characteristics (ROC) curves. Several texture parameters significantly differed between NSCLC and RP (p < 0.05). The parameters that were high in areas under the ROC curves (AUC) were as follows: SUV_max_, 0.64; GLRLM run percentage, 0.83 and NGTDM coarseness, 0.82. Diagnostic accuracy was improved using GLRLM run percentage or NGTDM coarseness compared with SUV_max_ (p < 0.01). The texture parameters of ^18^F-FDG uptake yielded excellent outcomes for differentiating NSCLC from radiation pneumonitis after CIRT, which outperformed SUV-based evaluation. In particular, GLRLM run percentage and NGTDM coarseness of ^18^F-FDG PET/CT images would be appropriate parameters that can offer high diagnostic accuracy.

## Introduction

Lung cancer is one of the most prevalent cancers worldwide, with non-small cell lung cancer (NSCLC) accounting for 85% to 90% of all forms of lung cancer^1^. Radiotherapy is an option for patients who are unable to tolerate or who decline surgery. Stereotactic body radiotherapy^2^ and particle therapy^3^ have been widely applied to such patients. Carbon‐ion radiotherapy (CIRT) has excellent dose‐localizing properties^4^, and can thus deliver a high dose to a target while avoiding adjacent critical organs at risk. The high radiosensitivity of the lungs constitutes a critical dose-limiting factor for treating thoracic tumors with radiation^5,6^. Various pulmonary side effects can arise after CIRT, such as radiographic lung damage, pleural reactions, pneumonitis, or fibrosis^7,8^.

Generally, CT cannot distinguish necrotic tumors or fibrotic scar tissues from residual or recurrent tumors^9^, which leads to a delay before undergoing repeat CIRT. On the other hand, metabolic imaging using ^18^F-fluorodeoxyglucose (^18^F-FDG) positron emission tomography/computed tomography (^18^F-FDG PET/CT) can discriminate between recurrence and post-treatment changes^10–12^, since ^18^F-FDG PET/CT can noninvasively indirectly measure glucose metabolism in vivo.

The maximum standardized uptake value (SUV_max_) is the most prevalent parameter used to estimate tumor metabolic activity in ^18^F-FDG PET/CT images. However, the SUV_max_ is measured as the most numerous pixels in a region of interest; images show only areas of the highest intensity of ^18^F-FDG uptake in a tumor and cannot reflect metabolic activity in whole tumors^13^. Therefore, the extent of active lesions in malignant tumors and active residual lesions after treatment is difficult to ascertain using SUV_max_ accurately. To overcome these issues, metabolic tumor volume (MTV) and total lesion glycolysis (TLG) have been proposed as supplementary diagnostic indicators of tumor activity^14^.

Few studies have discriminated lung cancer from radiation pneumonitis (RP) in images based on accumulated ^18^F-FDG. This suggests that ^18^F-FDG uptake after RT might be due not only to recurrent tumors but also to RT-induced inflammation. The characterization of uptake heterogeneity is gaining popularity through radiomics-based analyses that extract high throughput features based on intensity, shape, and the texture of uptake within regions of interest. Using textural features has improved the ability of ^18^F-FDG PET/CT to discriminate abnormal from normal tissues and delineate lesions. Textural features derived from Neighboring Gray Tone Difference Matrices describe features such as coarseness, contrast, and busyness on PET images^15^. They can differentiate cancerous tumors of the head and neck from normal tissues^16^. However, whether textural features can discriminate malignant from benign lesions remains unknown. The value of quantitative heterogeneity in ^18^F-FDG PET/CT images for RP after CIRT has not also been investigated. The aim of this study was to assess the ability of ^18^F-FDG PET/CT metabolic parameters and its textural image features to differentiate NSCLC from RP after CIRT to develop a differential diagnosis of malignancy and benign lesion.

## Methods

### Patients

We retrospectively analyzed ^18^F-FDG PET/CT data obtained from 32 patients with NSCLC who underwent ^18^F-FDG PET/CT before CIRT (50.0 GyE/day), and from 31 patients with RP who were diagnosed by biopsy or by clinical follow up at > 1 year after CIRT (50.0 GyE/day) for NSCLC. The NSCLC patients had not received chemotherapy before CIRT and underwent ^18^F-FDG PET/CT at baseline.

This clinical study was approved by the Ethics Committee at the National Institute of Radiological Sciences (Approval No. 19–019), and written informed consent was obtained from all patients. This study was conducted as a research involving research participants in accordance with the principles outlined in the 1964 Declaration of Helsinki and its later amendments. The results of this retrospective study did not influence further therapeutic decision-making.

### ^18^F***-FDG PET/CT***

Patients fasted for at least 6 h before being injected with 4 MBq/kg ^18^F-FDG. Whole-body images were acquired at a mean of 60 min later after voiding from the top of the skull to the midthigh using an Aquiduo PET/CT scanner (Canon Corp., Japan). Aquiduo PET/CT scanner has the following technical characteristics: detector material, Lu_2_SiO_5_(Ce) (LSO); crystal size, 4.0 × 4.0 × 20 mm^3^; detector ring diameter, 830 mm; transaxial field of view (FOV), 585 mm; axial FOV, 162 mm, coincidence window, 4.5 ns; energy window, 425–650 keV; maximum ring difference, 27; random correction, delayed; scatter correction, single scatter simulation. Emission data were acquired for 2–3 min per bed position^17^. The PET images were reconstructed using an iterative algorithm (the combination of Fourier rebinning and the ordered subsets expectation–maximization [FORE + 2D-OSEM]; 4 iterations, 14 subsets) with an 8-mm Gaussian filter, a 128 × 128 matrix (3.9 mm/pixel) and 81 slices (2 mm/slice). Time-of-flight and point spread function correction were not applied. The spatial resolution of this scanner according to NEMA NU 2–2007 is 6.5 mm in full width at half maximum (FWHM) at 10 mm off center. Whole-body spiral CT scanning proceeded under the following parameters: 120 kV; auto exposure control (noise level: SD 10); 512 × 512 matrix; beam pitch, 0.94; 2 mm × 16-row mode. The CT data were used for the attenuation correction.

### Quantitative analysis

We used the PET/CT medical imaging viewer, PETSTAT (AdIn Research Inc., Tokyo, Japan) for texture analysis. Volumes of interest (VOI) on tumors were delineated using a threshold of 40% (40P) of the SUV_max_ in each lesion. In the texture analysis of ^18^F-FDG PET/CT in NSCLC, 40P had excellent inter-operator reproducibility of texture features and has proven tolerable in previous studies^18^. It is also recommended for heterogeneity determination of lesions affected by respiratory migration^19^.

VOI was set not to include physiological accumulation. We calculated The SUV parameters of SUV_max_, SUV_peak_, SUV_mean_, MTV, TLG as well as fifty-six texture parameters derived from seven matrices for each VOI. SUV_peak_ is defined as the mean SUV in an 1 cm^3^ sphere around the pixel with the highest uptake and is assumed to be less affected by image noise than SUV_max_. For images with noise properties typically associated with clinical PET images, SUV_peak_ can provide a slightly more robust alternative. To extract the texture parameters, we first equalized histograms by rescaling the intensity within each ROI between the 1st and 99th percentiles of the ROI over 64 bins. Using 64 equally divided bins has been a common approach for image quantitation in radiomics analysis, and it also allows exploration of entire ranges of tumor signal intensity^20^.

### Statistical analysis

We compared all data between NSCLC and RP after CIRT using Wilcoxon rank-sum tests. Values lying nearest the upper left corner in ROC curves were considered to indicate optimal diagnostic accuracy. Sensitivity, specificity, and accuracy were calculated using appropriate cutoffs. Diagnostic accuracy was compared using areas under ROC curves (AUC), and ROC curves were also compared. All data were statistically analyzed using JMP v.14.2.0 software (SAS Institute Inc., Cary, NC, USA) and values with P < 0.05 were considered statistically significant.

## Results

### Characteristics of patients with NSCLC and RP

The 63 patients (age, 76.8 ± 8.2 years; male, n = 47 [74.6%]) included eight with squamous cell carcinoma (SCC), 33 with adenocarcinoma (ADC), 2 with neuroendocrine carcinoma, and 18 with unclassified non-small cell carcinoma. The sizes of the NSCLC and RP were 2.90 ± 1.59 (range, 71.9–19.0) and 5.08 ± 2.12 (range, 82.4–33.4) cm, respectively. Gender, and tumor size significantly differed between the NSCLC and RP groups (P < 0.05; Table 1).

**Table 1:** Characteristic of patients with NSCLC and RP.

Characteristics	NSCLC (n = 32)	RP (n = 31)	p value
Mean age (y)	76.8 ± 8.2	79.2 ± 7.7	0.322
**Gender**			
Male	24	23	
Female	8	8	
**Histology**			
Squamous cell carcinoma	6	4	
Adenocarcinoma	18	15	
Neuroendocrine carcinoma	1	1	
Unclassified non-small cell carcinoma	7	11	
Tumor size (cm)	2.90 ± 1.59	5.08 ± 2.12	< 0.0001*

*P < 0.05, statistically significant. NSCLC, non-small cell lung carcinoma; RP, radiation pneumonitis; SCC, squamous cell carcinoma.

### Comparison of NSCLC and RP

Statistical differences among parameters in the texture analysis were explored using Wilcoxon rank-sum tests (Table 2). Most texture parameters significantly differed between NSCLC and RP (P < 0.05), whereas most SUV parameters did not.

**Table 2:** Significant difference of each parameters.

Parameter	NSCLC (n = 32)	RP (n = 31)	p value
SUV_max_	4.95 ± 4.89	1.86 ± 0.71	0.063
SUV_peak_	4.31 ± 4.43	1.67 ± 0.62	0.084
SUV_mean_	2.96 ± 2.99	1.09 ± 0.41	0.080
MTV	8.64 ± 6.87	40.20 ± 25.84	< 0.0001*
TLG	31.11 ± 50.38	47.10 ± 35.27	0.001*
**GLRLM**			
Short Runs Emphasis mean	0.85 ± 0.32	0.92 ± 0.17	< 0.0001*
Short Runs Emphasis max	0.86 ± 0.32	0.94 ± 0.17	< 0.0001*
Long Runs Emphasis mean	0.98 ± 0.38	1.19 ± 0.22	< 0.0001*
Long Runs Emphasis max	1.04 ± 0.40	1.30 ± 0.26	< 0.0001*
Gray Level Nonuniformity mean	2.15 ± 2.38	7.78 ± 10.00	0.001*
Gray Level Nonuniformity max	2.30 ± 2.63	8.16 ± 10.46	0.006*
Run Length Nonuniformity mean	4.08 ± 4.09	14.83 ± 17.54	0.004*
Run Length Nonuniformity max	4.48 ± 4.69	16.08 ± 18.93	0.003*
Run Percentage mean	0.81 ± 0.31	0.84 ± 0.16	< 0.0001*
Run Percentage max	0.83 ± 0.32	0.88 ± 0.16	< 0.0001*
**GLSZM**			
High Intensity Emphasis	495.05 ± 224.06	473.08 ± 181.11	0.085
Low Intensity Emphasis	0.05 ± 0.02	0.04 ± 0.02	0.001*
Large Area Emphasis	1.20 ± 0.50	1.70 ± 0.38	< 0.0001*
Small Area Emphasis	0.68 ± 0.27	0.62 ± 0.13	< 0.0001*
Intensity Variability	1.43 ± 1.09	4.17 ± 4.54	0.012*
Run Length Variability	2.17 ± 1.41	5.64 ± 5.17	0.008*
Zone Percentage	0.65 ± 0.26	0.56 ± 0.12	< 0.0001*
**NGLCM3D**			
Uniformity mean	0.00 ± 0.00	0.00 ± 0.00	0.010*
Uniformity max	0.00 ± 0.00	0.00 ± 0.00	0.188
Entropy mean	5.44 ± 2.10	6.52 ± 1.21	0.001*
Entropy max	5.51 ± 2.13	6.70 ± 1.25	< 0.0001*
Dissimilarity mean	7.78 ± 3.55	5.42 ± 1.32	< 0.0001*
Dissimilarity max	9.96 ± 4.48	7.09 ± 2.00	< 0.0001*
Contrast mean	110.91 ± 64.96	50.31 ± 17.80	< 0.0001*
Contrast max	172.44 ± 97.36	81.64 ± 35.67	< 0.0001*
Homogeneity mean	0.17 ± 0.08	0.25 ± 0.05	< 0.0001*
Homogeneity max	0.23 ± 0.10	0.34 ± 0.07	< 0.0001*
Correlation mean	0.03 ± 0.02	0.01 ± 0.01	< 0.0001*
Correlation max	0.03 ± 0.03	0.01 ± 0.01	< 0.0001*
**NGLCM3DMean**			
Uniformity	0.00 ± 0.00	0.00 ± 0.00	0.318
Entropy	5.24 ± 2.01	6.05 ± 1.14	0.028*
Dissimilarity	4.27 ± 1.98	3.18 ± 0.88	< 0.0001*
Contrast	33.18 ± 20.16	20.06 ± 10.79	0.003*
Homogeneity	0.25 ± 0.11	0.36 ± 0.08	< 0.0001*
Correlation	0.04 ± 0.03	0.01 ± 0.01	< 0.0001*
**NGLCM**			
Uniformity mean	0.00 ± 0.00	0.00 ± 0.00	0.036*
Uniformity max	0.00 ± 0.00	0.00 ± 0.00	0.199
Entropy mean	5.42 ± 2.09	6.42 ± 1.20	0.001*
Entropy max	5.49 ± 2.11	6.59 ± 1.23	0.0003*
Dissimilarity mean	6.84 ± 3.12	4.60 ± 1.16	< 0.0001*
Dissimilarity max	8.43 ± 3.78	5.80 ± 1.54	< 0.0001*
Contrast mean	84.82 ± 49.77	35.32 ± 13.09	< 0.0001*
Contrast max	122.78 ± 69.72	53.65 ± 21.36	< 0.0001*
Homogeneity mean	0.18 ± 0.08	0.28 ± 0.06	< 0.0001*
Homogeneity max	0.22 ± 0.10	0.33 ± 0.07	< 0.0001*
Inverse Difference Moment mean	0.11 ± 0.06	0.19 ± 0.05	< 0.0001*
Inverse Difference Moment max	0.14 ± 0.07	0.24 ± 0.07	< 0.0001*
Correlation mean	0.03 ± 0.03	0.01 ± 0.01	< 0.0001*
Correlation max	0.03 ± 0.03	0.01 ± 0.01	< 0.0001*
**NGTDM**			
Coarseness	0.01 ± 0.00	0.00 ± 0.00	< 0.0001*
Contrast	0.00 ± 0.00	0.00 ± 0.00	< 0.0001*
Busyness	0.18 ± 0.14	0.80 ± 0.52	< 0.0001*
Complexity	45.72 ± 43.20	7.40 ± 4.85	< 0.0001*
Strength	6.30 ± 4.23	2.29 ± 1.89	< 0.0001*
**SUV Histogram**			
Variance	208.68 ± 88.57	195.13 ± 52.23	0.001*
Entropy	3.38 ± 1.29	3.73 ± 0.70	0.922

*P < 0.05, statistically significant.

### Diagnostic accuracy

**Figure 1:**
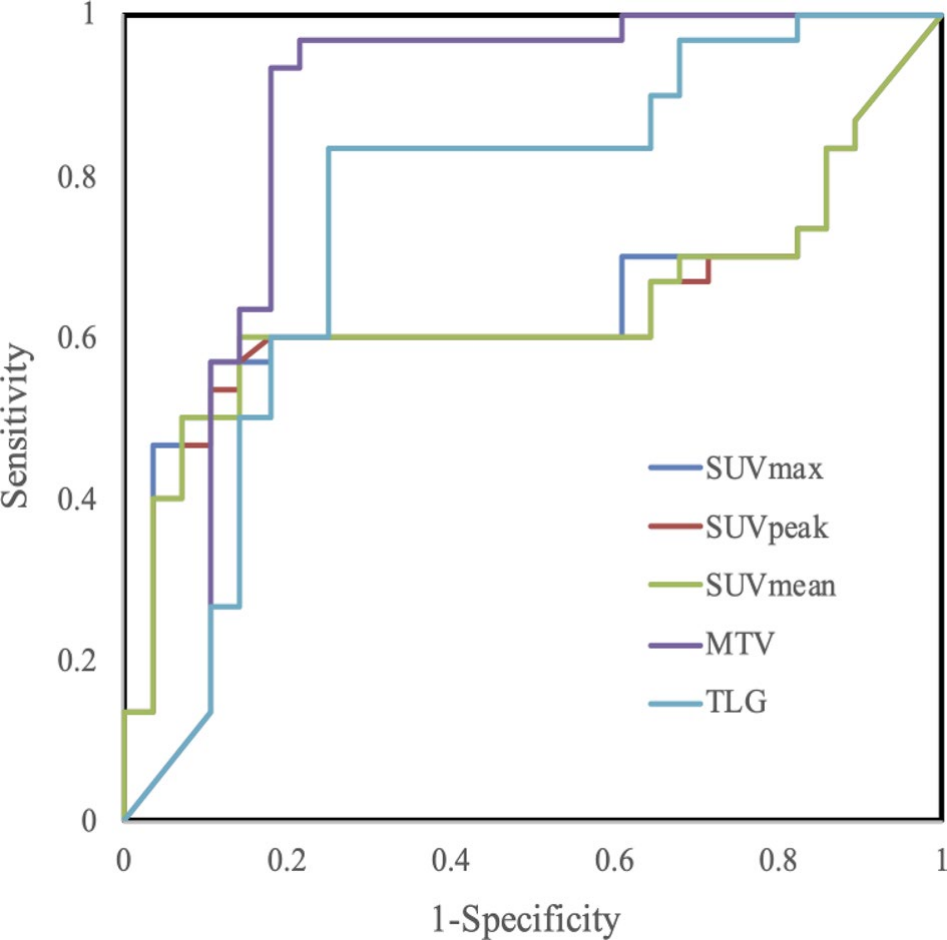
Receiver operating characteristic curves of ability of SUV parameters to discriminate NSCLC from RP.

**Figure 2:**
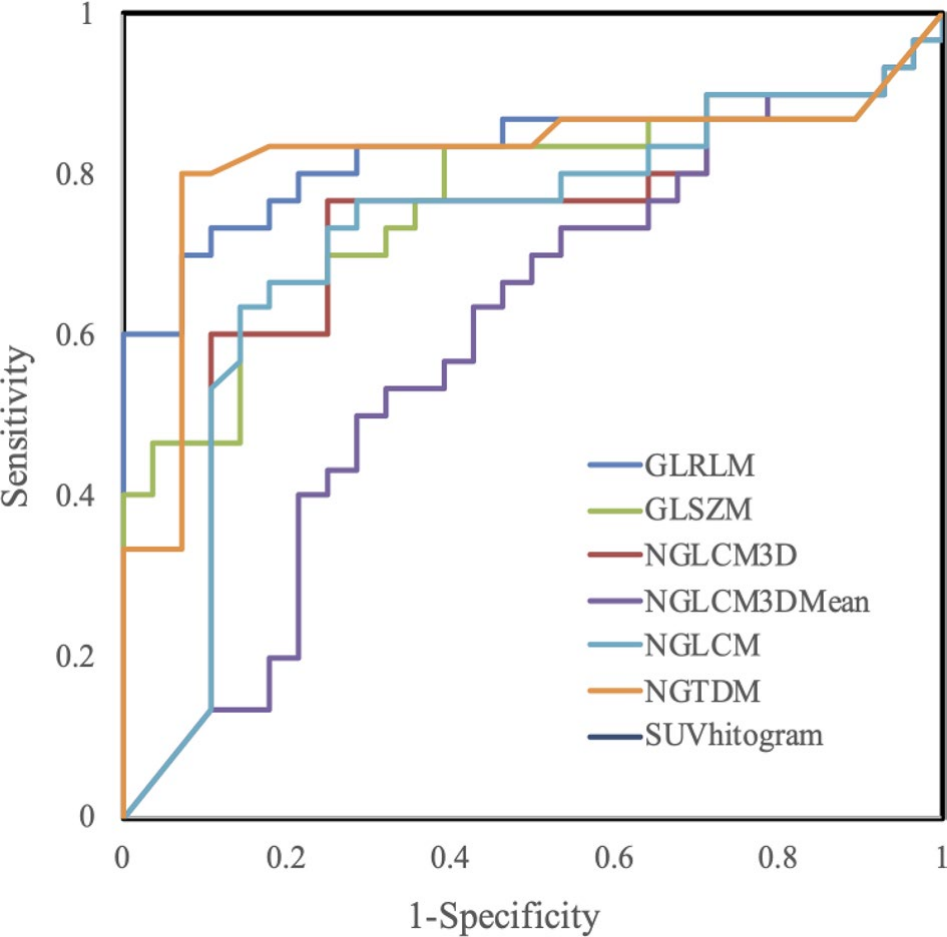
Receiver operating characteristic curves of ability of texture parameters to discriminate NSCLC from RP. GLRLM, gray-level run-length matrix run percentage; GLSZM, gray-level size-zone matrix intensity variability; NGLCM, normalized gray-level cooccurrence matrix dissimilarity; NGTDM, neighborhood gray-tone difference matrix coarseness; SUV Histogram, SUV histogram variance**.**

Figures 1 and 2 show the outcomes of ROC analyses for each parameter. The AUC of SUV_max_, SUV_peak_, MTV and TLG were respectively, 0.64, 0.63, 0.86 and 0.75. On the other hand, the AUC of the gray-level run-length matrix run percentage (GLRLM) and neighborhood gray-tone difference matrix coarseness (NGTDM) were 0.83 and 0.82, respectively, and significantly differed from the AUC of the SUV_max_ (Table 3).

**Table 3:** ^18^F-FDG PET/CT metrics and cutoffs for differentiation between NSCLC and RP.

Parameter	Cutoff	Sensitivity (%)	Specificity (%)	PPV (%)	NPV (%)	AUC
SUV_max_	2.80	56.67	10.71	85.00	65.79	0.64
SUV_peak_	2.64	53.33	10.71	84.21	64.10	0.63
SUV_mean_	1.54	60.00	14.29	81.82	66.67	0.63
MTV	16.37	93.33	17.86	84.85	92.00	0.86
TLG	27.88	83.33	25.00	78.13	80.77	0.75
GLRLM	0.43	70.00	7.14	91.30	74.29	0.83
GLSZM	0.16	60.00	14.29	81.82	66.67	0.76
NGLCM3D	0.17	76.67	25.00	76.67	75.00	0.71
NGLCM3DMean	0.24	50.00	28.57	65.22	57.14	0.59
NGLCM	0.15	63.33	14.29	82.61	68.57	0.72
NGTDM	0.00	80.00	7.14	92.31	81.25	0.82
SUV Histogram	0.92	40.00	3.57	92.31	60.00	0.65

*P < 0.05, statistically significant. AUC, area under ROC curve; GLRLM, gray-level run-length matrix run percentage; GLSZM, gray-level size-zone matrix intensity variability; NSCLC, non-small cell lung carcinoma; NGLCM, normalized gray-level cooccurrence matrix dissimilarity; NGTDM, neighborhood gray-tone difference matrix coarseness; NPV, negative predictive value; PPV, positive predictive value; RP, radiation pneumonitis; SUV Histogram, SUV histogram variance.

### Comparison of NSCLC and RP images

**Figure 3:**
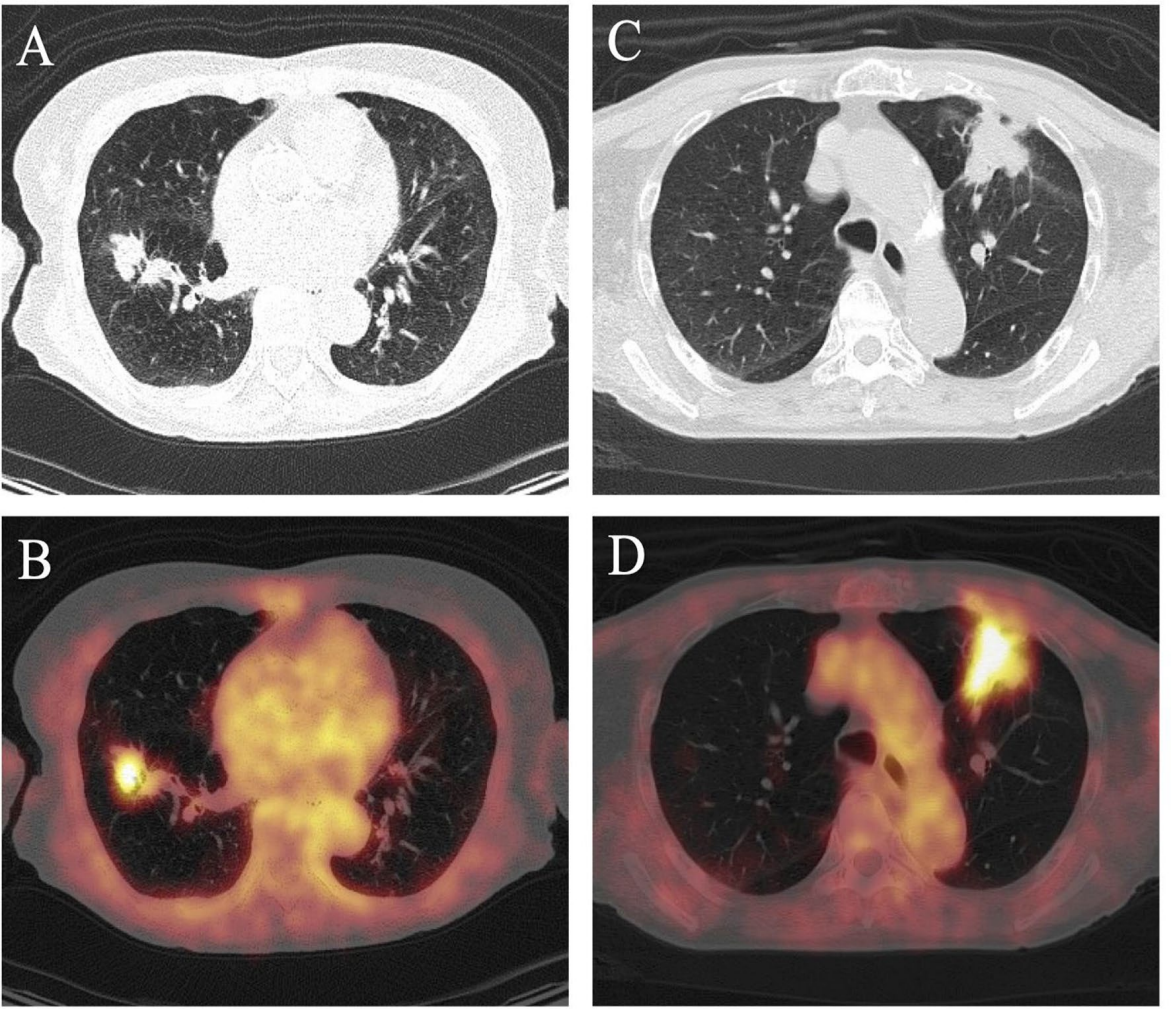
Representative CT and fused PET/CT images of NSCLC and RP. (**A**) CT image shows mass in right middle lobe of 79-year-old male with NSCLC. (**B**) Axial fused PET/CT image shows high ^18^F-FDG uptake (SUV_max_, 4.32) and heterogeneous ^18^F-FDG distribution (GLRLM run percentage, 0.81; NGTDM coarseness, 0.0068). (**C**) CT Image of 77-year-old male with RP shows mass-like attenuation in left upper lobe. (**D**) Axial fused PET/CT image shows high ^18^F-FDG uptake (SUV_max_, 3.75) and homogeneous ^18^F-FDG distribution (GLRLM run percentage, 0.65; GLSZM coarseness, 0.0017).

Figure 3 shows representative images. The GLRLM run percentage and NGTDM coarseness were significantly higher in NSCLC, than in RP, whereas SUV_max_ values of both lesions were similar.

## Discussion

To develop the differential diagnosis of malignancy and benign lesions, we compared whether texture parameters could more accurately differentiate NSCLC from RP in patients after CIRT than SUV parameters such as SUV_max_ and MTV. We found a possible relationship of textural imaging parameters for GLRLM run percentage and NGTDM coarseness of tumor heterogeneity measured on ^18^F-FDG PET/CT images, which would lead the ability to differentiate RP from NSCLC.

The MTV and TLG were significantly increased, whereas the SUV_max_ was reduced in RP compared with NSCLC. Buyger et al. reported that a fixed threshold could substantially underestimate TLG and MTV in lesions with high ^18^F-FDG uptake^21^. Therefore, a decrease in SUV_max_ might erroneously increase MTV because a larger volume of a less active tumor will be included in the MTV^22^. The SUV is not helpful for differentiating benign from malignant lesions^23^, and MTV and TLG are calculated based on SUV. Thus, we considered that MTV and TLG are not suitable for differentiating NSCLC and RP.

Almost all texture parameters significantly differed and had significantly better diagnostic ability than SUV_max_. In particular, the GLRLM run percentage and NGTDM coarseness would be appropriate parameters due to their high diagnostic accuracy. The GLRLM run percentage corresponded to the number of homogeneous runs of a specific intensity voxel within an image. We found that the GLRLM run percentage was significantly reduced in RP compared with NSCLC. This suggests that ^18^F-FDG accumulation is more heterogeneous in NSCLC than in RP. Hotta et al. reported that the GLRLM run percentage using a machine learning in^11^C-methionine PET images could distinguish recurrent brain tumors from radiation necrosis and that radiation necrosis was significantly reduced compared with recurrent brain tumors^24^. Adding the machine learning model might allow to create a model with higher sensitivity and specificity than this model proposed for benign and malignant lung differentiation. The NGTDM coarseness is based on differences between each voxel and neighboring voxels in adjacent image planes, and thus measures granularity within an image^25^. Chen et al. found significantly lower NGTDM coarseness in benign than malignant solitary pulmonary nodules^26^. The present findings revealed significantly lower NGTDM coarseness in RP than NSCLC, which is considered to reflect biological differences between benign and malignant lesions. Also, NGTDM coarseness is useful in discriminating benign and malignant cancers of the head and neck^27^.

The present findings of texture features are considered to reflect the heterogeneity of ^18^F-FDG accumulation in lesions. Several studies have found that the intratumoral heterogeneity of ^18^F-FDG uptake in tumors might be useful for evaluating therapeutic responses and predicting prognoses of NSCLC^20,28,29^, while there is a review paper by Han, S. et al., showing limited evidence to support the prognostic value of texture analysis in ^18^F-FDG PET in lung cancer^30^. Yet, little is understood about the application of ^18^F-FDG PET/CT texture analysis to the differential diagnosis of NSCLC and RP. The effects of CIRT on NSCLC can be highly beneficial**.** The application of ^18^F-FDG PET/CT texture analysis should improve the ability to distinguish NSCLC from RP. If so, then the amount of time required to determine a treatment regimen such as repeated irradiation could be decreased.

Most ^18^F-FDG PET/CT texture parameters (51 out of 56 parameters; Table 2) were useful for differentiating NSCLC from RP after CIRT. Gunma University has recently reported that SUV_max_ and MTV had better predictive or prognostic power for CIRT-treated NSCLC patients^31,32^. Metabolic information of ^18^F-FDG PET/CT imaging reflects a biological property of tumor as well as metabolically diverse components, including inflammatory tissues and surrounding normal tissues such as vessels, bronchi, and pleura after CIRT^29,33^. ^18^F-FDG PET/CT is considered to be playing key metabolic imaging in the management of CIRT for NSCLC.

This study had some limitations. Texture parameters could be affected by lesion size, histological tumor type, and inflammatory status^31^. The textural analysis also requires evaluation of so many variables, and the sample size is relatively small for this number of variables. Therefore, further studies in more patients with different types of tumors are needed. In addition, due to the limited spatial resolution of PET, image noise and partial volume effects may affect the results of this study^33^.

## Conclusion

We determined that texture parameters can differentiate NSCLC from RP after CIRT more accurately than SUV parameters such as SUV_max_ and MTV. The intratumoral heterogeneity of ^18^F-FDG uptake evaluated by texture analysis yielded improved diagnostic ability for differentiating NSCLC from RP after CIRT that outperformed SUV. In particular, GLRLM run percentage and NGTDM coarseness are appropriate parameters with high diagnostic accuracy. Our findings provide important information for understanding ^18^F-FDG PET/CT imaging in the management of CIRT for NSCLC.
